# Diterpenylquinone Hybrids: Synthesis and Assessment of Gastroprotective Mechanisms of Action in Human Cells

**DOI:** 10.3390/molecules180911044

**Published:** 2013-09-10

**Authors:** Cristina Theoduloz, Ivanna Bravo, Mariano Walter Pertino, Guillermo Schmeda-Hirschmann

**Affiliations:** 1 Laboratorio de Cultivo Celular, Facultad de Ciencias de la Salud, Universidad de Talca, Casilla 747, 3460000 Talca, Chile; 2 Laboratorio de Química de Productos Naturales, Instituto de Química de Recursos Naturales, Universidad de Talca, Casilla 747, 3460000 Talca, Chile; E-Mails: ibravo@utalca.cl (I.B.); mwalter@utalca.cl (M.W.P.); schmeda@utalca.cl (G.S.-H.)

**Keywords:** diterpenylquinones, hybrid compounds, gastroprotection, human cells, labdane diterpenes, lapachol

## Abstract

A modern approach in the search for new bioactive molecules is the synthesis of novel chemical entities combining molecules of different biosynthetic origin presenting biological effects as single compounds. Gastroprotective compounds from South American medicinal plants, namely quinones and diterpenes, were used as building blocks to obtain hybrid diterpenylquinones. Starting from the labdane diterpene junicedric acid and two isomers, as well as from three quinones, including lapachol, 18 hybrid molecules were synthesized. Six of them are described for the first time. The potential gastroprotective mechanisms of action of the compounds were assessed in dose-response experiments using human gastric epithelial cells (AGS) and human lung fibroblasts (MRC-5). The following studies were carried out: stimulation of cell proliferation, cytoprotection against sodium taurocholate (NaT)-induced damage, synthesis of PGE_2_ and total reduced sulfhydryl (GSH) content. The antioxidant capacity of the compounds was determined on the inhibition of the lipoperoxidation in human erythrocyte membranes. Hybrid compounds presented activities different from those shown by the starting compounds, supporting the potential of this approach in the search for new bioactive molecules. The effects might be modulated by selective modification in the terpene or quinone moieties of the new molecules. Structure-activity relationships are discussed.

## 1. Introduction

South American traditional medicine uses plants in therapeutic practice. Several herbal crude drugs have been shown to contain bioactive constituents that can be clearly related to their beneficial effects. The resin of the “araucaria” tree (*Araucaria araucana* (Mol.) K. Koch*,* Araucariaceae) was used by the Mapuche amerindians in Chile to treat gastric ulcers and to relieve stomach pain [1]. The stem bark of the crude drug “lapacho” or “taheebo” (*Tabebuia* spp., Bignoniaceae) was formerly used by the Guarani as an anti-inflammatory agent and then incorporated into Paraguayan folk medicine, to treat cancer and wounds [2]. The naphthoquinone lapachol from *Tabebuia* species [3] and labdane diterpenes from *A. araucana*, display gastroprotective activity *in vivo* [4] supporting the ethnobotanical information. The bioactive compounds from those crude drugs (quinones and diterpenes) exert their effect as single chemical entities. A new approach in drug design is to combine two different molecules with individual intrinsic effects into a single new hybrid compound [5]. Little has been done on the synthesis of products combining naturally occurring moieties arising from different biosynthetic pathways into such hybrid molecules. Recent studies presented the synthesis, gastroprotective effect and cytotoxicity of diterpenylnaphthoquinones [6] and the potential gastroprotective effect of novel sesquiterpene quinone derivatives in human cells [7]. Terpenylquinones have been described from some marine organisms and display several biological activities, including cytotoxicity and anti-inflammatory effect [8]. New hybrid compounds containing terpene and quinone moieties have been prepared and some of them present relevant biological effects including cytotoxicity [9,10] and antifungal effect [11].

The study of gastroprotective activity of compounds has been traditionally carried out using laboratory animals. However, due to social and economic pressures, the international trend has been a gradual reduction in the use of experimental animals. The use of primary cell culture as well as immortalized cell lines allows *in vitro* testing and studies of possible mechanisms of action. There are several advantages in using cell cultures as biological models, namely: the amount of compound is reduced to a minimum, variables are much better controlled than when working with entire animals and the costs are substantially reduced. More advanced techniques consider the use of gastric epithelial cells [7,12,13].

The aim of this work was to prepare new hybrid compounds using as starting molecules gastroprotective diterpenes with the labdane skeleton and quinones. Human cell cultures were used to assess their potential mechanisms of gastroprotective activity and compare it to that of the starting substances. To draw some structure-activity trends, the hybrids included variations both in the quinone as well as in the diterpene moieties.

## 2. Results and Discussion

### 2.1. Hybrid Compounds

**Scheme 1:**
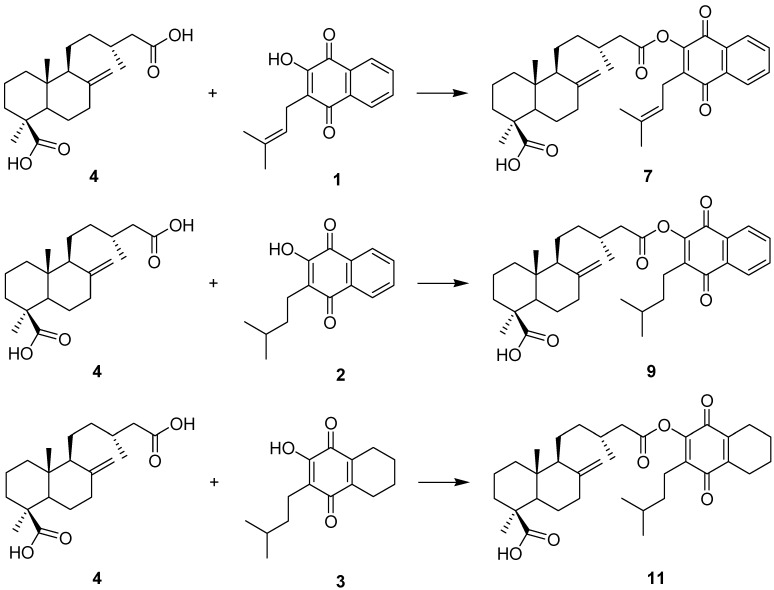
General synthetic procedures for preparation of the hybrid compounds. Quinones: compounds **1**, **2** and **3**. Diterpenes: compounds **4**, **5** and **6**.

**Figure 1:**
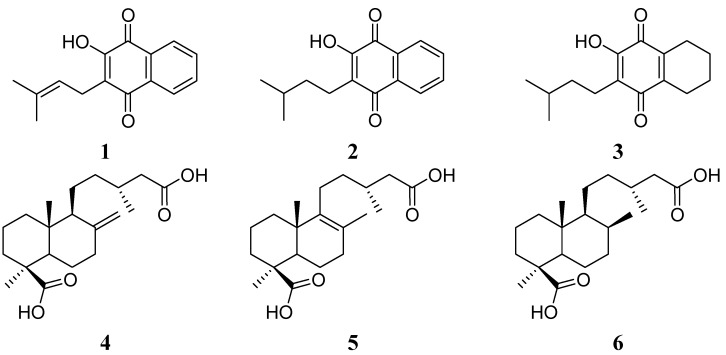
Structure of the starting quinones **1**–**3** and diterpenes **4**–**6**.

**Figure 2:**
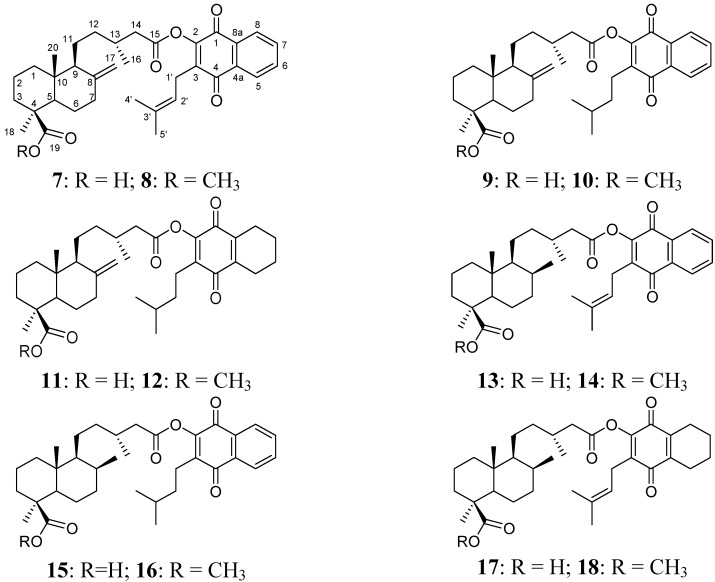
Diterpenylquinone derivatives of junicedric acid **7**–**12** and 17β-dihydrojunicedric acid **13**–**18**.

**Figure 3:**
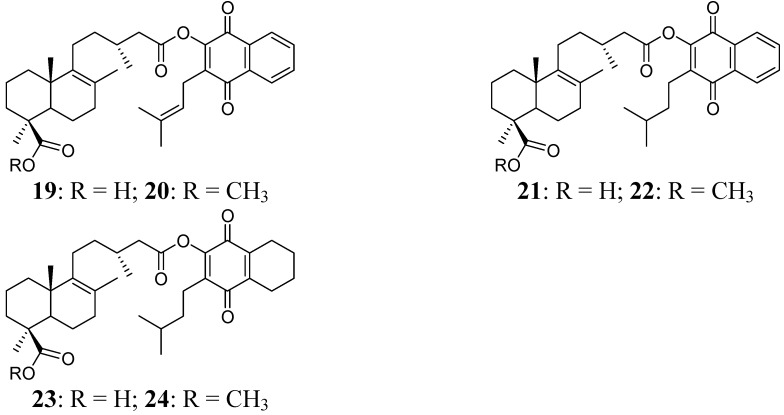
Diterpenylquinone derivatives of Δ8(9) junicedric acid **19**–**24**.

A series of hybrid compounds was synthesized using as building blocks diterpenes and quinones. The quinones included lapachol (**1**) and its hydrogenation products **2** and **3**. The quinone part of the molecule included an isoprenyl side chain, hydrogenated side chains with aromatic rings in the naphthoquinone moiety or hydrogenated side chains and aromatic rings. The diterpene junicedric acid (**4**) with an exocyclic double bond, its isomer **5** with an Δ8(9) double bond and the hydrogenation product **6** were used as the terpene part of the hybrid molecules. Eighteen hybrids, including six new compounds, were synthesized (Scheme 1), differing in some structural features while maintaining common characteristics to evaluate structure-activity trends. All compounds were characterized by spectroscopic and spectrometric means and are in agreement with the proposed structures. The structures of compounds **1**–**24** are shown in Figure 1, Figure 2 and Figure 3.

*Reagents and conditions*: (**a**): DCC, DMAP, CH_2_Cl_2_, rt; or (**b**): oxalyl chloride, CH_2_Cl_2_, quinone, TEA.

### 2.2. Cytotoxicity

The cytotoxicity of all compounds was evaluated on confluent cultures of human lung fibroblasts (MRC-5) and human gastric epithelial cells (AGS) to determine the working concentrations in the subsequent mechanism of action experiments. Cell viability was determined by means of the neutral red uptake assay and results are expressed as IC_50_ values (µM) in Table 1. Hydrogenation increases cytotoxicity for the lapachol derivatives **2** and **3**. From the starting diterpenes, Δ8(9) junicedric acid (**5**) presented lower toxicity towards MRC-5 and AGS cells, with IC_50_ values of 834 and 666 µM, respectively. The diterpene with higher cytotoxicity was compound **6** (IC_50_ values of 163 and 283 µM for MRC-5 and AGS cells, respectively).

Among the six hybrid compounds derived from diterpene **4** (compounds **7**–**12**), the carboxylic acid derivative **7** resulted more cytotoxic than the parent quinone and diterpene **1** and **4**, respectively. A similar trend was observed for hybrids **9** and **11**, prepared from quinones **2** and **3**, and diterpene **4**, respectively. Methylation of **7**, **9** and **11** led to the less cytotoxic derivatives of the series **8**, **10** and **12**, indicating that the free C-19 carboxylic acid function is relevant for the cytotoxicity of this series. The highest cytotoxicity value was found for compound **11**, in which both, the prenylated side chain and the benzene ring of the lapachol were reduced by hydrogenation.

The diterpene **5** differs from **4** in the position of the double bond, leading to different configurations for both compounds and their hybrid derivatives. Hybrid compounds containing diterpene **5** (compounds **19**–**24**), showed the same trend than derivatives from diterpene **4** when presenting a free C-19 COOH function. Therefore, compounds **19**, **21** and **23** were more toxic than their corresponding C-19 COOMe derivatives **20**, **22** and **24**. As for the series from diterpene **4**, the most cytotoxic compound for the derivative series of **5** resulted compound **23**, with the prenylated side chain and the benzene ring of the lapachol completely reduced.

Among the hybrid compounds obtained from diterpene **6** (compounds **13**–**18**), it was observed that hybrids **13** and **17** bearing a carboxylic acid function at C-19, were more toxic than the corresponding methylester derivatives **14** and **18**. However, when only hydrogenation of the prenylated side chain of the lapachol was present in the hybrid compounds (**15** and **16**), the ester derivative **16** showed higher cytotoxicity that the corresponding carboxylic acid derivative **15**.

**Table 1:** .Cytotoxicity expressed as IC_50_ values (µM) towards confluent cultures of human lung fibroblasts (MRC-5) and gastric epithelial cells (AGS) treated with the compounds **1**–**24** and lansoprazole.

Compound	IC_50_ ± SD (µM) *^a^*	
	MRC-5	AGS
Lapachol (**1**)	>1000	571 ± 28
Dihydroprenyl lapachol (**2**)	611 ± 42	292 ± 12
Dihydroprenyl-5,6,7,8-tetrahydrolapachol (**3**)	307 ± 22	87 ± 4
Junicedric acid (**4**)	439 ± 26	383 ± 23
Δ8(9) Junicedric acid (**5**)	834 ± 51	666 ± 35
17β-Dihydrojunicedric acid (**6**)	163 ± 5	283 ± 17
Lapachoyl junicedrate (**7**)	221 ± 13	143 ± 6
Lapachoyl junicedrate methyl ester (**8**)	>1000	>1000
Dihydroprenyl lapachol junicedrate (**9**)	449 ± 27	49 ± 10
Dihydroprenyl lapachoyl junicedrate methyl ester (**10**)	>1000	659 ± 42
Dihydroprenyl-5,6,7,8-tetrahydrolapachoyl junicedrate(**11**)	42 ± 2	63 ± 4
Dihydroprenyl-5,6,7,8-tetrahydrolapachoyl junicedrate methyl ester (**12**)	667 ± 40	>1000
Lapachoyl 17β-dihydrojunicedrate (**13**)	355 ± 21	351 ± 29
Lapachoyl 17β-dihydrojunicedrate methyl ester (**14**)	727 ± 48	916 ± 54
Dihydroprenyl lapachol 17β-dihydrojunicedrate (**15**)	>1000	>1000
Dihydroprenyl lapachol 17β-dihydrojunicedrate methyl ester (**16**)	596 ± 44	643 ± 39
Dihydroprenyl-5,6,7,8-tetrahydrolapachoyl 17β-dihydrojunicedrate (**17**)	44 ± 4	60 ± 5
Dihydroprenyl-5,6,7,8-tetrahydrolapachoyl 17β-dihydrojunicedrate methyl ester (**18**)	369 ± 29	884 ± 62
Lapachoyl Δ8(9) junicedrate (**19**)	129 ± 9	148 ± 9
Lapachoyl Δ8(9) junicedrate methyl ester (**20**)	483 ± 31	321 ± 24
Dihydroprenyl lapachoyl Δ8(9) junicedrate (**21**)	601 ± 26	278 ± 14
Dihydroprenyl lapachoyl Δ8(9) junicedrate methyl ester (**22**)	848 ± 51	340 ± 20
Dihydroprenyl-5,6,7,8-tetrahydrolapachoyl Δ8(9) junicedrate (**23**)	20 ± 2	29 ± 2
Dihydroprenyl-5,6,7,8-tetrahydrolapachoyl junicedrate methyl ester (**24**)	>1000	>1000
Lansoprazole *^b^*	316 ± 11	168 ± 8

Cells were treated during 24 h with the compounds. Cell viability was determined by means of the neutral red uptake assay. *^a^* Results are expressed as mean values ± SD. Each concentration was tested in quadruplicate together with the control and repeated three times in different experiments; *^b^* Reference compound.

### 2.3. MRC-5 Fibroblast Proliferation

**Figure 4:**
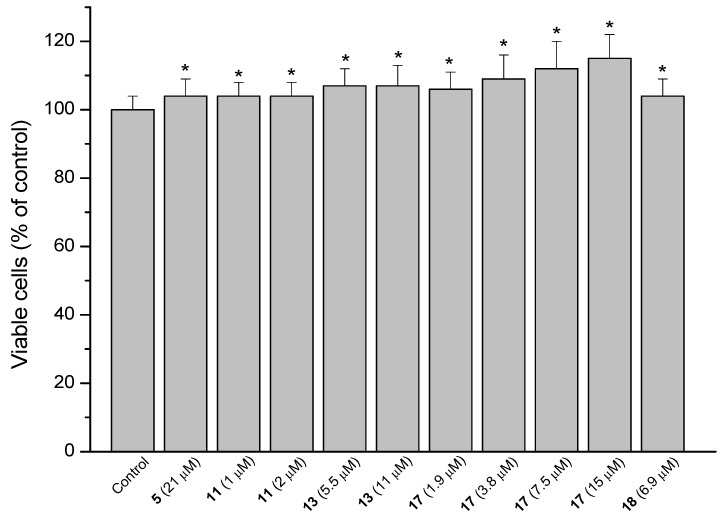
Stimulating effect of the diterpene **5** and derivatives **11**, **13**, **17** and **18** on the proliferation of MRC-5 cells. Each value represents the mean ± SD of three different experiments in quadruplicate. ANOVA followed by Dunnett´s test. * *p* < 0.05 compared to the control group.

A key factor in the renewal and repair of gastric mucosa is the proliferative capacity of fibroblasts [14]. The ability of the compounds to accelerate cell proliferation and hence gastric wound healing was determined using MRC-5 fibroblasts. The compounds were evaluated at six concentrations lower than the IC_50_ values, being IC_50_/2 the highest concentration tested. The positive results are presented in Figure 4. Percentual proliferative increase compared to untreated cells is shown in parentheses. Stimulation of fibroblast proliferation was observed for the diterpene **5** (3.6%), the hybrid **11** (3.6%), obtained by the combination of **4** and **3** and the derivatives from diterpene **6**: **13** (7.3%), **17** (5.5 to 14.5%) and **18** (3.6%). The active compounds **11**, **17** and **18** have in common the quinone **3** in their structures. These compounds differ from the inactive derivatives **23** and **24** (also containing the quinone **3**) by the more planar configuration of the diterpene moiety of compounds **23** and **24**. None of the hybrid derivatives that showed activity contain the diterpene moiety **5**.

### 2.4. Sodium Taurocholate (NaT)-Induced Damage

**Figure 5:**
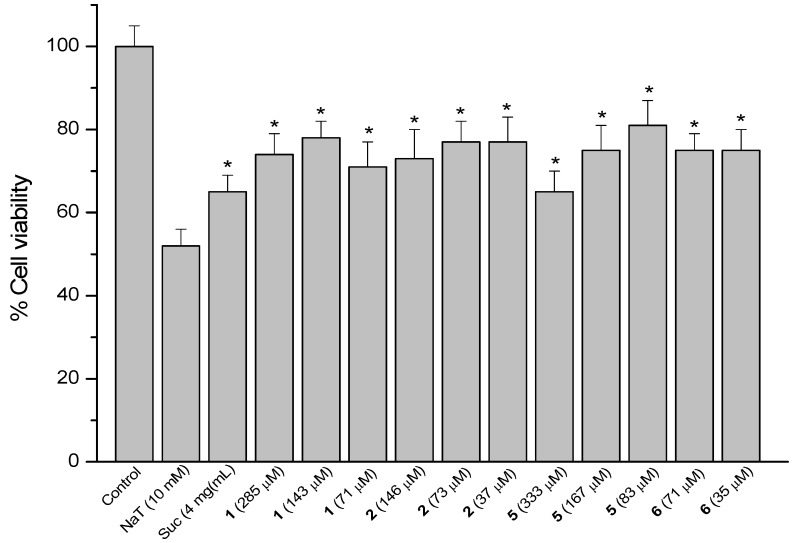
Effect of pre-treatment during 60 min with the reference compound sucralfate (Suc), the quinones **1** and **2** and the diterpenes **5** and **6** followed by an incubation during 30 min with 10 mM NaT on the viability of AGS cells determined by the neutral red uptake assay. Each value represents the mean ± SD of three different experiments in quadruplicate. ANOVA followed by Dunnett´s test. * *p* < 0.05 compared to NaT group.

**Figure 6:**
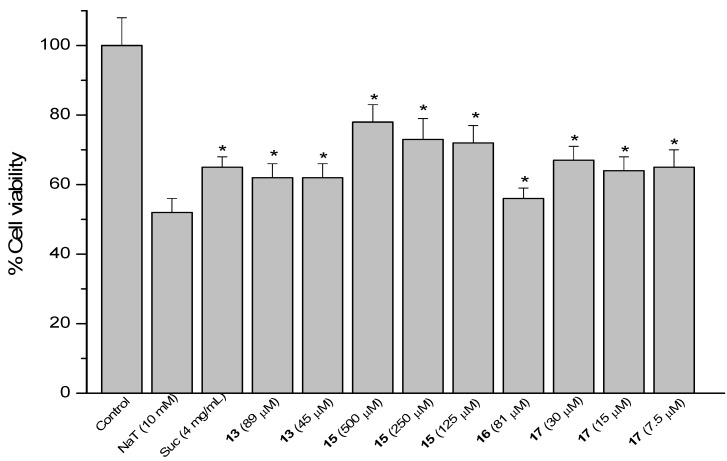
Effect of pre-treatment during 60 min with the reference compound sucralfate (Suc) and derivatives **13**, **15**, **16** and **17** from 17β-dihydrojunicedric acid, followed by an incubation during 30 min with 10 mM NaT on the viability of AGS cells determined by the neutral red uptake assay. Each value represents the mean ± SD of three different experiments in quadruplicate. ANOVA followed by Dunnett’s test. * *p* < 0.05 compared to NaT group.

**Figure 7:**
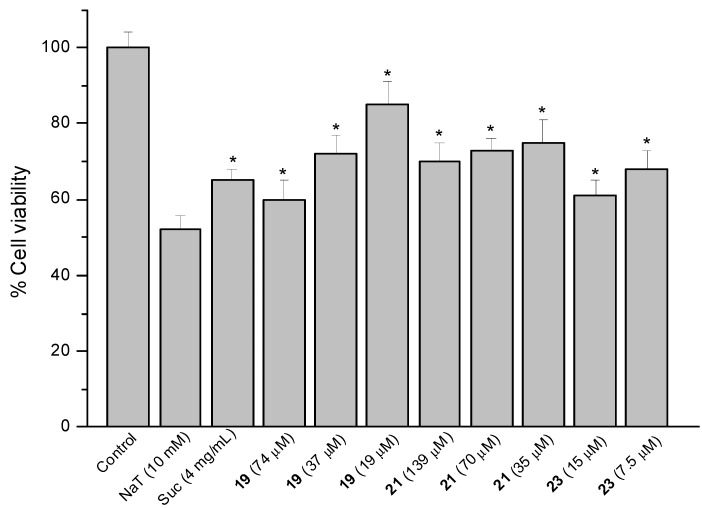
Effect of pre-treatment during 60 min with the reference compound sucralfate (Suc) and Δ8(9) junicedric acid derivatives **19**, **21** and **23** followed by an incubation during 30 min with 10 mM NaT on the viability of AGS cells determined by the neutral red uptake assay. Each value represents the mean ± SD of three different experiments in quadruplicate. ANOVA followed by Dunnett’s test. * *p* < 0.05 compared to NaT group.

The model of AGS cells damaged by NaT (bile salt) was used to assess the gastroprotective effect of compounds against the gastric ulcer induced by bile reflux [15]. Results are presented as a reduction in cell viability. Treatment of the cells with 10 mM NaT during 30 min caused a 48% reduction of cell viability compared with untreated controls. Cells were pre-treated during 60 min with the compounds at 1/2, 1/4 and 1/8 of the respective IC_50_ values and then 10 mM NaT was added to all wells. The reference compound sucralfate at 4 mg/mL (580 µM) showed a reduction of 35% in cell viability. The positive results are shown in Figure 5, Figure 6 and Figure 7.

The results showed significant cytoprotective effect (*p* < 0.05) for the starting quinones **1** (25, 22 and 29% cell protection at 285, 143 and 71 µM, respectively) and **2** (27, 23 and 23%, at 146, 73 and 37 µM respectively), the diterpenes **5** (35, 25 and 19% reduction in cell viability at 333, 167 and 83 µM, respectively) and **6** (25% cell viability reduction at 71 and 35 µM) (Figure 5).

Cytoprotection was observed for the diterpene **6** hybrids **13**, **15**–**17** (Figure 6). The effect of compound **17** at 30, 15 and 7.5 µM was in the same range as sucralfate (34%–36%), while the activity of **13** at 89 and 45 µM was 38% and the effect of **16** at 81 µM was 44%, compared to NaT controls. Best effect, even higher than that of sucralfate, was found for the hybrid **15** at 500, 250 and 125 µM, reducing cell viability by 21, 27 and 27%, respectively.

The hybrids of the diterpene **5** with **1**, **2** and **3** (compounds **19**, **21** and **23**) were active (Figure 7). However, the corresponding methyl esters were devoid of effect, suggesting that the free COOH at C-19 is needed for cytoprotection. Even if COOH is needed for cytoprotection, the carboxylic acid derivatives were the most toxic compounds. The cytoprotection effect might be due to the implication of OH residues in radical mechanisms with ROS species, and that might be the reason why the methyl ester derivatives, lacking of OH, do not present cytoprotective effects. Compound **19** reduced cell viability by 40, 27 and 15% at 74, 37 and 19 µM. Compound **21** at 139, 70 and 35 µM improved viability by 30, 27 and 25%, while the effect of compound **23** at 15 and 7.5 µM was 39 and 32%, respectively. The inverse dose-response effect of compounds **5** and **19** and in a less extent for **21** might be related to the solubility of the compounds.

Cytoprotective compounds may protect against NaT-induced damage forming a physical barrier or binding bile salts, interacting with cell membranes or modifying the expression of trefoil factor family 2 mRNA and *c-fos* protein [16,17].

### 2.5. Effect on Prostaglandin E_2_ (PGE_2_) Content

A relevant gastroprotective mechanism involves the prostaglandins that accelerate ulcer healing, epithelial cell proliferation, synthesis of growth factors and inhibition of inflammatory cell infiltration [18]. The prostaglandin E_2_ (PGE_2_) is considered to be the major humoral factor providing resistance to the gastric mucosa [19]. Some terpenes and their derivatives exert their gastroprotective effect increasing the gastric PGE_2_ synthesis *in vitro* and *in vivo* [7,20]. Experiments were carried out using post-confluent AGS cells. The results are presented in Table 2. Among the starting quinones and diterpenes **1**–**6**, only lapachol **1** stimulated PGE_2_ synthesis in AGS, cells showing a 2.5- and 2.3-fold increase in PGE_2_ content at 286 and 143 µM, respectively. Compound **8** at 500 and 250 µM elicited a 15.2- and 5.2-fold increase of PGE_2_ content, respectively.

**Table 2:** Effect of lapachol **1** and the diterpenyl naphthoquinones **8**–**10**, **15**, **18**, **21**, **22** and **24** on the total PGE_2_ content of post-confluent AGS cells treated during 1 h with the compounds at 1/2 and 1/4 of IC_50_.

Compound	Concentration (µM)	PGE_2_ (pg/mL)
Control	-	52 ± 5
Indomethacin *^a^*	100	Not detected
Lapachol (1)	286	132 ± 12 *
	143	117 ± 10 *
Lapachoyl junicedrate methyl ester (**8**)	500	787 ± 56 *
	250	272 ± 30 *
Dihydroprenyl lapachol junicedrate (**9**)	12.5	309 ± 29 *
Dihydroprenyl lapachoyl junicedrate methyl ester (**10**)	323	465 ± 39 *
	162	306 ± 32 *
Dihydroprenyl lapachol 17β-dihydrojunicedrate (**15**)	500	372 ± 25 *
	250	279 ± 20 *
Dihydroprenyl-5,6,7,8-tetrahydrolapachoyl 17β-dihydrojunicedrate methyl ester (**18**)	221	156 ± 13 *
	111	123 ± 9 *
Dihydroprenyl lapachoyl Δ8(9) junicedrate (**21**)	85	162 ± 18 *
Dihydroprenyl lapachoyl Δ8(9) junicedrate methyl ester (**22**)	170	114 ± 11 *
Dihydroprenyl-5,6,7,8-tetrahydrolapachoyl junicedrate methyl ester (**24**)	250	123 ± 12 *

Each value represents the mean ± SD of two different experiments in triplicate. ANOVA followed by Dunnett’s multiple comparison test. * *p* < 0.01 compared to control group. *^a^* Reference compound.

The dihydroprenyl lapachol hybrids **9**–**10**, **15**, **21**–**22**, bearing the same quinone moiety but differing in the identity of the terpene, increased PGE_2_ synthesis as follows: **9** (12.5 µM, 5.9-fold), **10** (323 and 162 µM, 8.9- and 5.9-fold), **15** (500 and 250 µM, 7.2 and 5.4-fold), **21** (85 µM, 3.1-fold) and **22** (170 µM, 2.2-fold), respectively.

PGE_2_ synthesis was also stimulated by compounds **18** (221 and 111 µM, 3.0 and 2.4-fold, respectively) and **24** (250 µM, 2.4-fold). The hybrids **18** and **24** have in common the quinone moiety **3** and differ in the identity of the diterpene part of the hybrid molecule.

### 2.6. Effect on Cellular Reduced Glutathione (GSH) Content

Generation of gastric ulcers has been related to the effect of free radicals. This fact explains the ulcerogenic effect of ethanol on the gastric mucosa. Intracellular GSH content is a determining factor that contributes to the protection of the gastric mucosa against oxidative stress [21]. Experiments were carried out using post-confluent AGS cells. The known stimulant of GSH synthesis, *N*-acetyl-L-cysteine (NAC) was used as positive control. Under our experimental conditions, NAC (750 µM) increased GSH content by 30% compared to untreated controls. Results are summarized in Table 3. Among the starting diterpenes, junicedric acid (**4**) and 17β-dihydrojunicedric acid (**6**) significantly (*p* < 0.05 and *p* < 0.01) stimulated GSH production. Best activity was observed for compound **6** (60 and 43% increase at 71 and 37 µM, respectively). Among the hybrid compounds, a good effect was found for the junicedric acid-lapachol derivatives **7** (23 and 18% at 36 and 18 µM, respectively) and **8** (24 and 19% at 250 and 125 µM, respectively) as well as for dihydroprenyl lapachol junicedrate **9** (16 and 15% at 12 and 6 µM, respectively).

Considering the diterpene **6 ** hybrids, best activity was found for the derivative **15** (35 and 31% at 250 and 125 µM, respectively), **17** (51 and 89% at 15 and 7.5 µM, respectively) and **18** (26% both at 221 and 111 µM). Compounds **17** and **18** differ in the free COOH (**17**) or COOCH_3_ function (**18**) in the diterpene moiety.

Most of the Δ8(9) junicedric acid (diterpene **5**) hybrids presented activity. However, the starting diterpene was inactive. In this group of compounds, effect was observed for the derivatives with lapachol (compound **20**, 13 and 19% at 80 and 40 µM, respectively), dihydroprenyl lapachol (compound **21**, 33% at 70 µM) and dihydroprenyl-5,6,7-8-tetrahydrolapachol (compound **23**, 26% at 7.3 µM). Inverse dose-response effect was observed for the hybrids **17**, **20** and **24**. This effect might be related to the solubility of the compounds.

**Table 3:** Total reduced sulfhydril (GSH) content in post-confluent AGS cells treated during 4 h with *N*-acetyl-L-cysteine (NAC) and the compounds **1**–**24** at 1/4 and 1/8 of IC_50_.

Compound	Concentration (µM)	GSH (nmol/10^6^ cells)
Control	-	13.6 ± 0.7
NAC*^a^*	750	* 17.7 ± 1.4
Lapachol(**1**)	143	14.1 ± 0.8
	72	13.9 ± 0.9
Dihydroprenyl lapachol(**2**)	73	13.8 ± 0.7
	37	13.6 ± 0.7
Dihydroprenyl-5,6,7,8-tetrahydrolapachol (**3**)	22	13.0 ± 0.5
	11	14.3 ± 0.7
Junicedric acid (**4**)	96	* 17.2 ± 1.0
	48	* 18.4 ± 1.1
Δ8(9) Junicedric acid (**5**)	166	14.1 ± 0.6
	83	13.6 ± 0.5
17β-Dihydrojunicedric acid (**6**)	71	** 21.7 ± 1.3
	37	* 19.5 ± 0.9
Lapachoyl junicedrate (**7**)	36	* 16.7 ± 0.7
	18	16.1 ± 0.9
Lapachoyl junicedrate methyl ester (**8**)	250	* 16.8 ± 0.9
	125	* 16.2 ± 0.6
Dihydroprenyl lapachol junicedrate (**9**)	12	* 15.8 ± 0.6
	6	* 15.6 ± 0.6
Dihydroprenyl lapachoyl junicedrate methyl ester (**10**)	165	13.0 ± 0.7
	83	13.4 ± 0.7
Dihydroprenyl-5,6,7,8-tetrahydrolapachoyl junicedrate (**11**)	16	13.2 ± 0.5
	8	13.1 ± 0.6
Dihydroprenyl-5,6,7,8-tetrahydrolapachoyl junicedrate methyl ester (**12**)	250	13.9 ± 0.8
	125	13.7 ± 1.0
Lapachoyl 17β-dihydrojunicedrate (**13**)	88	11.4 ± 0.6
	44	11.9 ± 0.4
Lapachoyl 17β-dihydrojunicedrate methyl ester (**14**)	229	12.7 ± 0.4
	115	14.4 ± 0.5
Dihydroprenyl lapachol 17β-dihydrojunicedrate (**15**)	250	* 18.4 ± 1.2
	125	* 17.8 ± 0.9
Dihydroprenyl lapachol 17β-dihydrojunicedrate methyl ester (**16**)	160	14.1 ± 0.6
	80	14.9 ± 0.7
Dihydroprenyl-5,6,7,8-tetrahydrolapachoyl 17β-dihydrojunicedrate (**17**)	15	** 20.5 ± 1.0
	7.5	** 25.8 ± 1.6
Dihydroprenyl-5,6,7,8-tetrahydrolapachoyl 17β-dihydrojunicedrate methyl ester (**18**)	221	* 17.2 ± 1.0
	111	* 17.1 ± 1.1
Lapachoyl Δ8(9) junicedrate (**19**)	37	13.5 ± 0.5
	19	13.1 ± 0.4
Lapachoyl Δ8(9) junicedrate methyl ester (**20**)	80	* 15.4 ± 1.3
	40	* 16.2 ± 1.0
Dihydroprenyl lapachoyl Δ8(9) junicedrate (**21**)	70	* 18.1 ± 1.1
	35	13.3 ± 0.7
Dihydroprenyl lapachoyl Δ8(9) junicedrate methyl ester (**22**)	85	14.2 ± 0.6
	43	13.6 ± 0.9
Dihydroprenyl-5,6,7,8-tetrahydrolapachoyl Δ8(9) junicedrate (**23**)	7.3	* 17.1 ± 1.3
	3.6	12.6 ± 0.8
Dihydroprenyl-5,6,7,8-tetrahydrolapachoyl junicedrate methyl ester(**24**)	250	* 18.0 ± 0.9
	125	* 18.7 ± 1.0

*^a^* NAC: reference compound. Each value represents the mean ± SD of three different experiments in triplicate. ANOVA followed by Dunnet’s test. * *p* < 0.05; ** *p* < 0.01.

### 2.7. Inhibition of Lipoperoxidation

Antioxidant effect of the compounds was assessed using human erythrocyte membranes. The products were tested at 100 µg/mL and results are presented in Table 4. Among the starting quinones, the hydrogenation products of lapachol **2** and **3** were more active than lapachol (**1**), with IC_50_ values of 25.4 and 33.3 µg/mL, respectively. From the diterpene moieties, only Δ8(9)-junicedric acid (**5**) was active with an IC_50_ of 43.8 µg/mL. The inhibition of lipoperoxidation for the hybrids prepared from the diterpene **4** (compounds **7**–**12**) was moderate. Better effects were found for the diterpene **4** derivative with lapachol and a COOCH_3_ at C-19 (**8**, IC_50_: 81.4 µg/mL), and the hybrids containing **4** and the quinone **2**, either with a free C-19 carboxylic acid (**9**, IC_50_: 40.1 µg/mL) or as the methyl ester (**10**, IC_50_: 99.4 µg/mL). Among the hybrids of diterpene **6** (compounds **13**–**18**), best antioxidant activity was found for: **13**, **14** and **15** with IC_50_ values of 61.1, 19.7 and 25.3 µg/mL, respectively. The three compounds contain the quinone **1** or **2** in the hybrid structure. The compounds **19**–**24** were prepared using Δ8(9) junicedric acid (**5**) as the diterpene moiety and presented moderate antioxidant effect, best activity was displayed by **20** (IC_50_: 58.1 µg/mL) and **23** (IC_50_: 59.5 µg/mL). In the same assay, the reference compound catechin presented an IC_50_ value of 75.4 µg/mL.

Structural diversity is a requirement to draw structure-activity relationships/trends in compounds series. The mechanisms of action of the starting diterpenes and quinones were compared with those of the new hybrids. The starting compounds showed activity in one or two (exceptionally three) out of the mechanisms of action investigated. Diterpene **5** stimulates cell proliferation, while none of its hybrids presented effect. From the four hybrids of diterpenes **4** and **6** with the quinone **3**, three were active.

The quinones **1**–**2** and diterpenes **5**–**6** were active in the NaT assay. Hybrids from diterpenes **5** and **6** with quinones show effects on NaT when the COOH group at C-19 of the diterpene is free (compounds **13**, **15**, **17**, **19**, **21** and **23**). The corresponding methyl esters (except compound **16**) were devoid of effect. The products prepared from the diterpene **4**, with a 8(17) double bond were inactive.

Among the parent molecules, only lapachol increased PGE_2_ synthesis. Relevant changes in PGE_2_ levels were observed for hybrid compounds, with several products stimulating synthesis. Regardless of the diterpene moiety, hybrids with dihydroprenyl lapachol **2** and two methyl esters from the hybrids with **3** were active.

In the GSH assay, quinones were inactive, but the diterpenes **4** and **6** presented effect. For the hybrids of diterpene **4**, the activity is observed for the products with a planar naphthoquinone (**7**–**9**) but it is lost when the aromatic ring of the quinone is hydrogenated. The diterpene **5**, with a double bond Δ8(9) has a more planar configuration and was devoid of activity. However, four out of the six hybrids from **5** proved to be active. Most of them presented the free COOH function at C-19.

**Table 4:** Effect of compounds **1**–**24** on the inhibition of the lipoperoxidation in human erythrocyte membranes. *^a^* Percent effect at 100 µg/mL or IC_50_ values (µg/mL).

Compound	% Inhibition of the lipoperoxidation *^a^*
Lapachol (**1**)	38
Dihydroprenyl lapachol (**2**)	IC_50_ 25.4 ± 2.4 µg/mL
Dihydroprenyl-5,6,7,8-tetrahydrolapachol (**3**)	IC_50_ 33.3 ± 2.3 µg/mL
Junicedric acid (**4**)	25
Δ8(9) Junicedric acid (**5**)	IC_50_ 43.8 ± 2.8 µg/mL
17β-dihydrojunicedric acid (**6**)	30
Lapachoyl junicedrate (**7**)	10
Lapachoyl junicedrate methyl ester (**8**)	IC_50_ 81.4 ± 11.0 µg/mL
Dihydroprenyl lapachol junicedrate (**9**)	IC_50_ 40.1 ± 5.3 µg/mL
Dihydroprenyl lapachoyl junicedrate methyl ester (**10**)	IC_50_ 99.4 ± 9.1 µg/mL
Dihydroprenyl-5,6,7,8-tetrahydrolapachoyl junicedrate (**11**)	44
Dihydroprenyl-5,6,7,8-tetrahydrolapachoyl junicedrate methyl ester (**12**)	37
Lapachoyl 17β-dihydrojunicedrate (**13**)	IC_50_ 61.1 ± 5.9 µg/mL
Lapachoyl 17β-dihydrojunicedrate methyl ester (**14**)	IC_50_ 19.7 ± 1.4 µg/mL
Dihydroprenyl lapachol 17β-dihydrojunicedrate (**15**)	IC_50_ 25.3 ± 4.1 µg/mL
Dihydroprenyl lapachol 17β-dihydrojunicedrate methyl ester (**16**)	46
Dihydroprenyl-5,6,7,8-tetrahydrolapachoyl 17β-dihydrojunicedrate (**17**)	48
Dihydroprenyl-5,6,7,8-tetrahydrolapachoyl 17β-dihydrojunicedrate methyl ester (**18**)	28
Lapachoyl Δ8(9) junicedrate (**19**)	49
Lapachoyl Δ8(9) junicedrate methyl ester (**20**)	IC_50_ 58.1 ± 4.1 µg/mL
Dihydroprenyl lapachoyl Δ8(9) junicedrate (**21**)	Nd
Dihydroprenyl lapachoyl Δ8(9) junicedrate methyl ester (**22**)	46
Dihydroprenyl-5,6,7,8-tetrahydrolapachoyl Δ8(9) junicedrate (**23**)	IC_50_ 59.5 ± 4.3 µg/mL
Dihydroprenyl-5,6,7,8-tetrahydrolapachoyl junicedrate methyl ester (**24**)	27
Catechin *^b^*	IC_50_ 75.4 ± 6.0 µg/mL

Results are expressed as mean values ± SD of three different experiments in triplicate. *^b^* Reference compound. Nd: not determined due to turbidity.

Among the starting compounds, the quinones **2** and **3** as well as the diterpene **5**, displayed inhibitory activity in the lipoperoxidation assay. Best antioxidant effect was found for hybrids from the diterpene **6** either with the quinone **1** or **2** or with weaker effect for diterpene **6** hybrids with the same quinones.

In a previous report, part of the compounds presented in this study, were evaluated for gastroprotective effect by the HCl-EtOH-induced gastric lesions model in mice [6].The compounds were administered orally at a single dose of 5 mg/kg. Compounds **9**–**10** and **21**–**22** displayed significant activity *in vivo* [6]. In the present study, these compounds stimulated the PGE_2_ synthesis in AGS cells. Our results show that compounds **8** and **20**, active *in vivo*, increased GSH content in AGS cells and protected erythrocyte membranes against induced lipoperoxidation. These *in vitro* findings sustain the *in vivo* observations suggesting that different and complementary mechanisms may be underlying the gastroprotective effect of the compounds.

Gastric lesions are a consequence of an imbalance between defensive and aggressive factors in the gastric mucosa. Increase in the defensive factors, namely: restitution of gastric epithelium, protection against damage induced by bile salts and free radicals, increase in PGE_2_ levels, GSH, bicarbonate and others, may reduce the appearance of gastric lesions in animals [7,16,17]. It has been shown that gastroprotective compounds exert their effects mainly stimulating the defensive factors in the gastric mucosa rather than inhibiting the aggressive agents (gastric acid or pepsin) [16,17,22].

In this work the possible mechanisms of gastroprotective effect of diterpenylquinones were assessed using human lung fibroblasts (MRC-5) and gastric epithelial cells (AGS). The evaluation of the compound activity on some defensive factors depending on gastric epithelial cells has been carried out using AGS cells. This cell line has been successfully used in the study of different aspects related to the gastric mucosa [7,23,24]. The hybrid compounds investigated stimulated one or more of the defensive factors described as relevant to protect the gastric mucosa. To obtain hybrid molecules with better activity than the parent compounds, structural modifications should be undertaken, followed by a suitable combination of substituents placed at the appropriate positions of hybrid compounds [25]. To understand the relationship between *in vitro* and *in vivo* activity, further research should be carried out, since factors such as absorption, bioavailability, location of the targets in different tissues or organs, receptor and enzyme densities will influence in effect [26].

## 3. Experimental

### 3.1. General Experimental Procedures

Optical rotations were obtained for solutions in CHCl_3_ (concentrations expressed in g/100 mL) on a Jasco DIP 370 polarimeter (Jasco Analytical Instruments, Easton, MD, USA). IR spectra were recorded on a Nicolet Nexus FT-IR instrument (Thermo Electron Corporation, Waltham, MA, USA).^1^H-NMR spectra were recorded at 400 MHz and ^13^C-NMR data were obtained at 100 MHz on a Bruker Avance spectrometer (Bruker, Rheinstetten, Germany). Chemical shifts are given in δ (ppm) with TMS as the internal standard. Mass spectra were measured in a LC/MSD-TOF (Agilent Technologies) by the electrospray technique. Positive or negative ion mode was used according to the samples. As internal reference in the ESI (+) mode, purine (*m/z* 121.0509) and HP-0921 (*m/z* 922.0098) were used. In the negative mode: ESI (−), purine (*m/z* 112.9856) and HP-0921 (*m/z* 1033.9881) were used as internal references. Silica gel 60 (Merck, 63-200 µm particle size) was used for column chromatography, precoated silica gel plates (Merck, Kieselgel 60 F254, 0.25 mm) were used for thin layer chromatography (TLC). TLC spots were visualized by spraying the chromatograms with p-anisaldehyde-ethanol-acetic acid-H_2_SO_4_(2:170:20:10 v/v) and heating at 110 °C for 3 min. 1,3-Dicyclohexylcarbodiimide (DCC) and dimethylaminopyridine (DMAP) were from Merck (Schuchardt, Germany). Oxalyl chloride was from Sigma-Aldrich (St. Louis, MO, USA).

### 3.2. General Procedure for the Synthesis of Hybrid Compounds

#### 3.2.1. Quinones

The naphthoquinone lapachol (**1**) was isolated from the wood of “lapacho” tree (*Tabebuia* sp). The hydrogenation products of lapachol (compounds **2** and **3**) were prepared trating (**1**) in EtOAc with Pd/C [6]. The synthetic scheme for the synthesis is presented in [6].

#### 3.2.2. Diterpenes

The diterpene **4** was obtained from the resin of *Araucaria araucana* as previously reported [6]. The isomers **5** and **6** were prepared from **4**. Briefly, treatment of **4** with HBr in acetic acid afforded Δ8(9) junicedric acid (**5**) while catalytic hydrogenation of **4** in ethyl acetate with 10% Pd/C in a 1:10 molar ratio yielded the hydrogenation product **6**. The synthetic scheme for the synthesis is shown in [6].

#### 3.2.3. Hybrid Compounds

The compounds **7**–**14** and **19**–**22** were synthesized according to [6]. The new compounds **15** and **17** were prepared by treating the diacid **6** (1 mEq) in dry CH_2_Cl_2_ (DCM) with 1,3-dicyclohexylcarbodiimide (DCC) (1 mEq) at room temperature under constant stirring. After 10 min, the quinones **2** and **3** (1 mEq) dissolved in dry DCM, were added together with a catalytic amount of dimethylaminopyridine (DMAP). After 2–4 h, the reaction was stopped by adding water and extracted with DCM. Compound **23** was synthesized from the diacid **5** under an inert (N_2_) atmosphere. Briefly, the diterpene **5** was dissolved in dry CH_2_Cl_2_ (DCM) and ice cooled under nitrogen flow. To this solution, oxalyl chloride in dry DCM was added drop wise with stirring. The diterpene: oxalyl chloride molar ratio was 1:40. The mixture was stirred at room temperature overnight, then the DCM was evaporated and the residue vacuum dried. The dry residue was dissolved in dry DCM and the quinone **3** was added as well as triethylamine (TEA) under constant N_2_ flow. The diterpene acyl chloride-quinone-TEA molar ratio was 1:1:2. The reaction mixture was left at room temperature under stirring and inert atmosphere for two days. Then, the mixture was washed two times with water, and the aqueous phase extracted with DCM to obtain the crude reaction mixture.

The reaction mixtures were dried over Na_2_SO_4_. Purification was carried out using a combination of gel permeation in Sephadex LH-20 with a MeOH:DCM 1:1 (*v/v*) mixture, or silica gel column chromatography using PE:EtOAc mixtures (90:10, 85:15, 80:20) (*v/v*), to afford compounds **15**, **17** and **23** in 14.3, 13.7 and 17% w/w yields, respectively. The methyl esters **16**, **18**, and **24** were obtained treating the compounds **15**, **17** and **23** with diazomethane in diethyl ether, with 92%–98% w/w yield. The purity of all the derivatives was over 95%, as determined by ^1^H-NMR spectroscopy. The new compounds **15**–**18**, **23** and **24** are described below.

*Dihydroprenyl lapachol 17β-dihydrojunicedrate* (**15**): 17β-Dihydrojunicedric acid (**6**, 246.8 mg, 0.732 mmol), DCC (302 mg, 1.464 mmol), a catalytic amount of DMAP and dihydroprenyl lapachol (**2**, 177.9 mg, 0.732 mmol), were stirred at room temperature in dry CH_2_Cl_2_ (20 mL) for 2–4 h. The reaction mixture was cooled in an ice bath. After addition of water, the aqueous phase was extracted with EtOAc (3 × 20 mL). The extract was dried over anhydrous Na_2_SO_4_ and taken to dryness under reduced pressure. Purification was carried out using a combination of gel permeation in Sephadex LH-20 with a MeOH-DCM 1:1 (*v/v*) mixture and silica gel column chromatography using PE-EtOAc mixtures 90:10 (*v/v),* to afford compound **15** (60.7 mg, 14.3%): yellow resin; 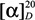 +2.35 (*c* 0.085 CHCl_3_); IR *ν*_max_ (film) 2957, 2931, 2854, 1725, 1675, 1463, 1386, 1287, 1074, 998 cm^−1^; ^1^H-NMR (CDCl_3_): δ 8.11 (2H, m, quinone H-5 and H-8), 7.75 (2H, m, quinone H-6 and H-7), 2.74 (1H, dd, *J* = 16, 4 Hz, H-14 α), 2.55 (2H, t, *J* = 8 Hz, H-1'), 2.42 (1H, m, H-14 β), 1.61 (1H, m, H-3'), 1.45-1.52 (2H, m, H-2'), 1.27 (3H, s, H-18), 1.13 (3H, d, *J* = 6.8 Hz, H-16), 0.93 (6H, d, *J* = 6.4 Hz, H-4' and H-5'), 0.90 (3H, d, *J* = 6.4 Hz, H-17), 0.84 (3H, s, H-20); ^13^C-NMR (CDCl_3_): 39.61 (C-1), 19.43 (C-2), 37.45 (C-3), 45.46 (C-4), 52.82 (C-5), 23.00 (C-6), 34.94 (C-7), 29.00 (C-8), 57.64 (C-9), 38.84 (C-10), 18.84 (C-11), 35.12 (C-12), 30.79 (C-13), 40.72 (C-14), 170.29 (C-15), 20.10 (C-16), 15.17 (C-17), 28.04 (C-18), 178.08 (C-19), 14.89 (C-20); Quinone: 173.42, 184.49 (C-1 and C-4), 151.04 (C-2), 140.09 (C-3), 130.89, 132.08 (C-4a, C-8a), 126.61, 126.50 (C-5 and C-8), 133.95, 133.72 (C-6 and C-7), 22.40 (C-1'), 37.00 (C-2'), 28.34 (C-3'), 22.28 (C-4'), 22.28 (C-5'); EIMS *m/z* 576 [M]^+^ (2), 293 (17), 244 (20), 243 (20), 242 (100), 227 (27), 123 (28), 109 (14), 95 (10), 81 (11), 55 (11).

*Dihydroprenyl lapachol 17β-dihydrojunicedrate methyl ester* (**16**). Yellow resin; 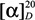 +20 (*c* 0.056, CHCl_3_); IR *ν*_max_ (film) 2951, 2868, 2844, 1772, 1725, 1679, 1463, 1380, 1300, 1151, 1081, 941, 712 cm^−1^; ^1^H-NMR (CDCl_3_): δ 8.11 (2H, m, quinone H-5 and H-8), 7.74 (2H, m, quinone H-6 and H-7), 3.65 (3H, s, OCH_3_), 2.75 (1H, dd, *J* = 14.8, 5.2 Hz, H-14 α), 2.55 (2H, t, *J* = 8 Hz, H-1'), 2.44 (1H, dd, *J* = 15.2, 8.8 Hz, H-14 β), 1.61 (1H, m, H-3'), 1.45-1.52 (2H, m, H-2'), 1.19 (3H, s, H-18), 1.13 (3H, d, *J* = 6.4 Hz, H-16), 0.95 (6H, d, J = 6.8 Hz, H-4' and H-5'), 0.92 (3H, d, *J* = 7.6 Hz, H-17), 0.70 (3H, s, H-20); ^13^C-NMR (CDCl_3_): 40.23 (C-1), 19.47 (C-2), 38.55 (C-3), 44.31 (C-4), 53.31 (C-5), 23.43 (C-6), 35.35 (C-7), 29.66 (C-8), 57.89 (C-9), 39.08 (C-10), 19.28 (C-11), 35.62 (C-12), 31.27 (C-13), 41.18 (C-14), 170.79 (C-15), 20.57 (C-16), 15.34 (C-17), 29.26 (C-18), 178.51 (C-19), 14.68 (C-20), 51.55 (OCH_3_); Quinone: 173.26, 184.95 (C-1 and C-4), 151.5 (C-2), 140.54 (C-3), 131.32, 132.52 (C-4a, C-8a), 127.06, 126.95 (C-5 and C-8), 134.41, 134.18 (C-6 and C-7), 22.86 (C-1'), 37.90 (C-2'), 28.79 (C-3'), 22.74 (C-4'), 22.74 (C-5'); molecular formula: C_36_H_50_O_6_ (578.3607). *m/z* 596.3947 [M+NH_4_]^+^, error: 0.20 ppm with the empirical formula C_36_H_54_NO_6_; *m/z* 601.3499 [M+Na]^+^, error: 0.08 ppm with the empirical formula C_36_H_50_O_6_Na.

*Dihydroprenyl-5,6,7,8-tetrahydrolapachoyl 17β-dihydrojunicedrate* (**17**). 17β-Dihydrojunicedric acid (**6**, 229.2 mg, 0.679 mmol), DCC (280.2 mg, 1.358 mmol), a catalytic amount of DMAP and dihydroprenyl-5,6,7,8-tetrahydrolapachol (**3**, 168.4 mg, 0.679 mmol), were stirred at room temperature in dry CH_2_Cl_2_ (20 ml) for 2–4 h. The reaction mixture was cooled in an ice bath. After addition of water, the aqueous phase was extracted with EtOAc (3 × 20 mL). The extract was dried over anhydrous Na_2_SO_4_ and taken to dryness under reduced pressure. The residue was purified by silica gel column chromatography, eluting with an hexane/EtOAc gradient (95:5, 91:9, 87:13), yielding **17** (52.9 mg, 13.7%): amber resin; 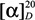 +23 (*c* 0.065, CHCl_3_); IR *ν*_max_ (film) 2959, 1769, 1699, 1662, 1625, 1549, 1467, 1455, 1382, 1269, 1205, 1077, 913, 755 cm^−1^; ^1^H-NMR (CDCl_3_): δ 2.68 (1H, dd, *J* = 14.8, 5.2 Hz, H-14 α), 2.40 (2H, m, quinone H-5 and H-8), 2.28 (1H, m, H-14 β), 1.70 (2H, m, quinone H-6 and H-7), 1.59 (2H, m, H-1'), 1.48 (2H, m, H-2'), 1.25 (3H, s, H-18), 1.10 (3H, d, *J* = 6.8 Hz, H-16), 0.92 (6H, d, *J* = 6.8 Hz, H-4' and H-5'), 0.92 (3H, d, *J* = 6.4 Hz, H-17), 0.80 (3H, s, H-20); ^13^C-NMR (CDCl_3_): 40.14 (C-1), 19.22 (C-2), 38.31 (C-3), 44.20 (C-4), 53.34 (C-5), 23.41 (C-6), 35.36 (C-7), 29.67 (C-8), 57.86 (C-9), 39.30 (C-10), 18.33 (C-11), 35.60 (C-12), 31.24 (C-13), 41.13 (C-14), 170.97 (C-15), 20.51 (C-16), 15.36 (C-17), 29.45 (C-18), 180.70 (C-19), 14.79 (C-20); Quinone: 184.36, 187.37 (C-1 and C-4), 149.02 (C-2), 141.29 (C-3), 137.32, 143.20 (C-4a, C-8a), 23.22, 21.45 (C-5 and C-8), 22.30, 21.45 (C-6 and C-7), 23.41 (C-1'), 37.90 (C-2'), 28.67 (C-3'), 22.71 (C-4'), 22.71 (C-5'); molecular formula: C_35_H_52_O_6_ (568.3764). *m/z* 586.4086 [M+NH_4_]^+^, error: 0.20 ppm with the empirical formula C_35_H_56_NO_6_.

*Dihydroprenyl-5,6,7,8-tetrahydrolapachoyl 17β-dihydrojunicedrate methyl ester* (**18**). Yellow resin; 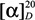 +43 (*c* 0.014 g/100 mL CHCl_3_); IR *ν*_max_ (film) 2956, 2846, 1772, 1729, 1662, 1464, 1452, 1385, 1208, 1193, 1151, 1077, 910 cm^−1^; ^1^H-NMR (CDCl_3_): 21 δ 3.57 (3H, s, OCH_3_), 2.60 (1H, dd, *J* = 14.8, 5.2 Hz, H-14 α), 2.31 (2H, m, quinone H-5 and H-8), 2.30 (1H, m, H-14 β), 2.07 (1H, d, *J* = 12.4 Hz, H-13), 1.62 (2H, m, quinone H-6 and H-7), 1.53 (2H, m, H-1'), 1.10 (3H, s, H-18), 1.01 (3H, d, *J* = 6.8 Hz, H-16), 0.84 (6H, d, *J* = 6.8 Hz, H-4' and H-5'), 0.82 (3H, d, *J* = 7.2 Hz, H-17), 0.60 (3H, s, H-20); ^13^C-NMR (CDCl_3_): 40.22 (C-1), 19.46 (C-2), 38.55 (C-3), 44.32 (C-4), 53.31 (C-5), 23.42 (C-6), 35.34 (C-7), 29.65 (C-8), 57.89 (C-9), 39.08 (C-10), 19.27 (C-11), 35.62 (C-12), 31.26 (C-13), 41.13 (C-14), 170.97 (C-15), 20.52 (C-16), 15.33 (C-17), 29.26 (C-18), 178.53 (C-19), 14.66 (C-20), 51.54 (OCH_3_); Quinone: 180.73, 187.37 (C-1 and C-4), 149.03 (C-2), 141.30 (C-3), 137.33, 143.21 (C-4a, C-8a), 23.07, 21.45 (C-5 and C-8), 22.30, 21.45 (C-6 and C-7), 23.23 (C-1'), 37.91 (C-2'), 28.68 (C-3'), 22.70 (C-4'), 22.70 (C-5'); molecular formula: C_36_H_54_O_6_ (582.3920). *m/z* 600.4242 [M+NH_4_]^+^, error: 2.85 ppm with the empirical formula C_36_H_58_NO_6_.

*Dihydroprenyl-5,6,7,8-tetrahydrolapachoyl* *Δ8(9) junicedrate* (**23**). Δ8(9) Junicedric acid (**5**, 228 mg, 0.678 mmol), oxalyl chloride (3.44 mg, 27.12 mmol), dihydroprenyl lapachol (**2**, 0.168 mg, 0.678 mmol) and triethylamine (TEA) (0.137 mg, 1.356 mmol) were dissolved in dry CH_2_Cl_2_ (DCM) under nitrogen flow. The reaction mixture was left at room temperature under stirring and inert atmosphere for two days. Then, the mixture was washed two times with water, and the aqueous phase extracted with DCM to obtain the crude reaction mixture. The extracts were dried over Na_2_SO_4_. Purification was carried out using silica gel column chromatography using PE:EtOAc 90:10 (v/v), to afford compound **23 **(65.4 mg, 17%): orange resin; 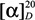 +31 (*c* 0.036, CHCl_3_); IR *ν*_max_ (film) 2954, 2864, 1772, 1695, 1662, 1619, 1469, 1380 1263, 1207, 1134, 1114, 1077, 911 cm^−1^; ^1^H-NMR (CDCl_3_): δ 2.61 (1H, dd, *J* = 15.3, 5.5 Hz, H-14 α), 2.41 (4H, m, quinone H-5 and H-8), 2.40–2.35 (1H, m, 14 β), 2.40-2.33 (2H, m, H-1'), 2.08 (1H, m, H-13), 1.67 (4H, m, quinone H-6 and H-7), 1.56 (3H, s, H-17), 1.53 (1H, m, H-3'), 1.30 (2H, m, H-2'), 1.24 (3H, s, H-18), 1.08 (3H, d, *J* = 6.6 Hz, H-16), 0.89 (3H, d, *J* = 6.6 Hz, H-4'), 0.89 (3H, d, J = 6.6, H-5'), 0.85 (3H, s, H-20), ^13^C-NMR (CDCl_3_): 37.22 (C-1), 19.89 (C-2), 37.38 (C-3), 44.18 (C-4), 53.92 (C-5), 21.45 (C-6), 37.16 (C-7), 127.18 (C-8), 139.35 (C-9), 40.13 (C-10), 26.13 (C-11), 34.62 (C-12), 31.75 (C-13), 41.35 (C-14), 170.87 (C-15), 19.88 (C-16), 20.11 (C-17), 29.04 (C-18), 180.68 (C-19), 18.29 (C-20); Quinone: 184.73, 187.36 (C-1 and C-4), 149.01 (C-2), 141.29 (C-3), 137.32, 143.21 (C-4a, C-8a), 23.22, 22.86 (C-5 and C-8), 21.45, 21.11 (C-6 and C-7), 22.28 (C-1'), 37.49 (C-2'), 28.66 (C-3'), 22.71 (C-4'), 22.71 (C-5'); molecular formula: C_35_H_50_O_6_ (566.3607), *m/z* 567.3687 [M+H]^+^, error: 1.11 ppm with the empirical formula C_35_H_51_O_6_; *m/z* 584.3944 [M+NH_4_]^+^, error: 0.23 ppm with the empirical formula C_35_H_54_NO_6_.

*Dihydroprenyl-5,6,7,8-tetrahydrolapachoyl* *Δ8(9) junicedrate methyl ester* (**24**). Yellow solid; 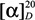 +22 (*c* 0.027, CHCl_3_); IR *ν*_max_ (film) 2953, 2868, 1769, 1726, 1659, 1470, 1449, 1379, 1138, 1081, 916, 761 cm^−1^; ^1^H-NMR (CDCl_3_): δ 3.61 (3H, s, OCH_3_), 2.76 (1H, dd, *J* = 15.3, 5.5 Hz, H-14 α), 2.62 (1H, dd, *J* = 15.3, 8.1 Hz, H-14 β), 2.06 (1H, m, H-13), 2.40 (4H, m, quinone H-5 and H-8), 2.40-2.33 (2H, m, H-1'), 1.67 (4H, m, quinone H-6 and H-7), 1.56 (3H, s, H-17), 1.53 (1H, m, H-3'), 1.27 (2H, m, H-2'), 1.18 (3H, s, H-18), 1.07 (3H, d, *J* = 6.8 Hz, H-16), 0.89 (3H, d, *J* = 6.4 Hz, H-4'), 0.89 (3H, d, J = 6.4, H-5'), 0.75 (3H, s, H-20), ^13^C-NMR (CDCl_3_): 37.63 (C-1), 19.98 (C-2), 37.89 (C-3), 44.28 (C-4), 53.93 (C-5), 21.25 (C-6), 37.62 (C-7), 127.18 (C-8), 139.42 (C-9), 39.95 (C-10), 26.12 (C-11), 34.71 (C-12), 31.75 (C-13), 41.36 (C-14), 170.88 (C-15), 19.89 (C-16), 20.14 (C-17), 28.86 (C-18), 178.55 (C-19), 18.14 (C-20), 51.51 (OCH_3_); Quinone: 180.69, 187.36 (C-1 and C-4), 149.00 (C-2), 141.28 (C-3), 137.32, 143.21 (C-4a, C-8a), 23.22, 22.71 (C-5 and C-8), 21.44, 21.44 (C-6 and C-7), 22.28 (C-1'), 38.13 (C-2'), 28.66 (C-3'), 22.84 (C-4'), 22.84 (C-5'); molecular formula: C_36_H_52_O_6_ (580.3764). *m/z* 581.3832 [M+H]^+^, error: 0.65 ppm with the empirical formula C_36_H_53_O_6_; *m/z* 598.4102 [M+NH_4_]^+^, error: 0.13 ppm with the empirical formula C_36_H_56_NO_6_.

### 3.3. Cell Culture

Human lung fibroblasts MRC-5 (ATCC CCL-171) were grown in minimum essential Eagle medium (MEM) with Earles’s salts and 2 mM L-glutamine (Sigma-Aldrich Co.). Human epithelial gastric cells AGS (ATCC CRL-1739) were grown in Ham F-12 medium containing 1 mM l-glutamine. Both media were supplemented with 1.5 g/L sodium bicarbonate (Sigma-Aldrich Co.), 10% heat-inactivated fetal bovine serum (FBS), 100 IU/mL penicillin and 100 µg/mL streptomycin.Both cell lines were grown as monolayers in a humidified incubator with 5% CO_2_ in air at 37 °C. Culture media, antibiotics and FBS were obtained from Invitrogen Corp.

### 3.4. Cytotoxicity Assay

Cytotoxicity values were required as a reference to determine the working concentrations in the subsequent experiments. Briefly, confluent cultures of MRC-5 and AGS cells were treated during 24 h with medium containing the compounds at concentrations ranging from 0 up to 1,000 µM. The products were first dissolved in DMSO (1% final concentration) and then in the corresponding culture medium supplemented with 2% FBS. Untreated cells were used as controls. Cell viability was determined at the end of the incubation by means of the neutral red uptake (NRU) assay [27]. Lansoprazole (Sigma-Aldrich Co., min. 98% by TLC) was used as reference compound.

### 3.5. MRC-5 Fibroblast Proliferation Assay

One day after seeding, cells were treated during 4 days with medium supplemented with 10% FBS and the studied compounds at concentrations ranging from 1/64 up to 1/2 of the respective IC_50_ values. Untreated cells were used as controls. Cell viability was determined at the end of the incubation by means of the NRU assay [27].

### 3.6. Sodium Taurocholate-Induced Damage to AGS Cells

The effect of sodium taurocholate (NaT) on cell viability was determined according to [15]. Briefly, one day post-confluent AGS cells were incubated during 60 min with the compounds at 1/2, 1/4 and 1/8 of the respective IC_50_ values. Then, 10 mM NaT was added to all wells for 30 min. Untreated cells were used as controls. Sucralfate (4 mg/mL; 580 µM) was used as reference compound (Sigma-Aldrich Co., min. 30% sucrose octasulfate by HPLC). After incubation, the NRU assay was carried out [27].

### 3.7. Determination of Prostaglandin E2 (PGE2) Content

One day after confluence, AGS cells were treated for 1 h with the compounds at 1/2 and 1/4 of the respective IC_50_ values. A control with medium only was included. Indomethacin (100 μM) was used as standard inhibitor of prostaglandin synthesis (Sigma-Aldrich Co., min. 99% by TLC). After incubation, PGE_2_ content was determined by means of a specific enzyme immunoassay kit (R & D Systems KGE004B, Minneapolis, MN, USA) and values were calculated according to the manufacturer instructions. Results are expressed as pg/well.

### 3.8. Determination of Cellular Reduced Glutathione (GSH) Content

One day after confluence, AGS cells were incubated during 4 h with the compounds at 1/4 and 1/8 of their respective IC_50_ values. Untreated cells were used as controls. The GSH synthesis stimulant *N*-acetyl-l-cysteine (750 µM) was used as reference substance (Sigma-Aldrich Co., ≥ 90% by HPLC). After incubation, the GSH content was determined using a colorimetric kit (BioAssays Systems, Hayward, CA, USA). Results are expressed as nanomol of soluble reduced sulfhydryls/10^6^ cells.

### 3.9. Inhibition of Lipoperoxidation in Erythrocyte Membranes

The inhibition of lipid peroxidation was determined using human erythrocyte membranes [28]. The compounds were tested at 100 µg/mL. Catechin was used as the reference compound (Sigma-Aldrich Co., min 98% by TLC).

### 3.10. Statistical Analysis

Results were expressed as mean values ± SD. Experiments with MRC-5 and AGS cells were carried out three times using different cell preparations. Each concentration was tested in quadruplicate. Statistical differences between different treatments and their respective control were determined by one-way analysis of variance (ANOVA) followed by the Dunnett’s multiple comparison test. The level of significance was set at *p* < 0.05 and *p* < 0.01. All statistical analyses were carried out using the software SPSS 12.0 for Windows.

## 4. Conclusions

The aim of this work was to synthesize new diterpenylquinones, to assess the possible mechanisms of the gastroprotective effects of the hybrid compounds using cell cultures and to establish some structure-activity trends. The hybrid compounds displayed activities different from those shown by the starting compounds, supporting the potential of this approach in the search for new bioactive molecules. These effects were modulated by selective modification in the terpene or quinone moieties of the hybrids. The changes in effect associated to the stereochemistry of the diterpenes (*i.e.*, a more flexible configuration for diterpenes with a 8(17) double bond or the 17-β-dihydroderivative *vs.* the Δ8(9) isomer) or with modifications in the planar configuration of the napththoquinones, suggest that the different mechanisms involved might include interactions with active sites of enzymes and/or receptors. Molecular modeling can be a useful tool for better understanding the clues behind the observed effects.
